# Production of an extract rich in alpha-tomatine from green tomatoes by subcritical water

**DOI:** 10.1098/rsos.250240

**Published:** 2025-06-11

**Authors:** Catarina Faria-Silva, Bruno Pedras, Nuno Costa, Manuela Carvalheiro, Sandra Simões, Pedro Simões

**Affiliations:** ^1^ Research Institute for Medicines (iMed.ULisboa), Faculty of Pharmacy, Universidade de Lisboa, Lisbon, Portugal; ^2^ LAQV-REQUIMTE, Department of Chemistry, NOVA School of Science and Technology, Universidade Nova de Lisboa, Portugal

**Keywords:** tomatoes, alpha-tomatine, subcritical water extraction

## Abstract

Green tomatoes, a by-product of agro-food industry, are rich in the glycoalkaloid alpha-tomatine. This compound presents health benefits including anti-inflammatory and fungicide properties. Subcritical water extraction (SWE), a green and sustainable process, was used to obtain a tomatine-rich extract from fresh or frozen tomatoes. SWE extracted *ca* 200 mg tomatine/100 g tomato, an amount higher than conventional methods, at a temperature of 190°C and a residence time of 15 min. Green tomatoes' SWE extracts were characterized in terms of their composition and antioxidant activity. The phenolic content obtained was approximately 200 mg of gallic acid equivalents/100 g tomatoes, and the saponin content was 1000 mg of tomatine equivalents/100 g tomatoes. Total carbohydrate content was different between fresh and frozen tomatoes, 1812 mg of D(+)- glucose equivalents/100 g tomatoes versus 1269 mg/100 g, respectively. In terms of antioxidant activity, a value around 100 mg of Trolox equivalents/100 g of tomatoes was obtained in the 2,2-diphenyl-1-picrylhydrazyl assay, whereas a value of 558 mg of Trolox equivalents/100 mg fresh tomatoes versus 452 mg of Trolox equivalents/100 g frozen tomatoes was obtained by cupric ion reducing antioxidant capacity assay. SWE extraction proved to be a valuable method to extract glycoalkaloids from green tomatoes. The obtained extracts have the potential to be used as ingredients and actives in the cosmetic and pharmaceutical industries.

## 1. Introduction

Tomato belongs to the *Solanaceae lycopersicum* species and is a rich source of several nutrients. During industrial tomato harvest, green and red tomatoes are separated by a colour-sorting machine, with the green tomatoes being returned to the fields as they hold no industrial value [1]. In Portugal, approximately 112 thousand tonnes of green tomatoes are discarded per year [2]. Nowadays, there is a trend in the agro-food industry to recover and find better uses for all their by-products [3]. Green tomatoes are particularly rich in glycoalkaloids, whose content decreases as the fruit ripens [4]. Glycoalkaloids are a group of steroidal alkaloids with a sugar attached, responsible for protecting the plant against bacteria, fungi, viruses and insects [5–7]. The glycoalkaloid present in all parts of the tomato plant is tomatine, a mixture of two glycoalkaloids—alpha-tomatine and dehydrotomatine—in a 10 : 1 ratio [1,8]. Alpha-tomatine offers numerous health-promoting activities, including antiviral, fungicidal, antibiotic, anti-inflammatory, anticarcinogenic, anti-obesity and anti-ageing [1,6,9,10]. Additionally, tomatoes are rich in phenolic compounds, which are associated with their antioxidant capacity [3]. In recent years, the use of ingredients from renewable plant sources or extracted from leftover materials has opened a new chapter in cosmetics and cosmeceuticals development [11,12].

Tomatine extraction techniques have traditionally involved conventional processes, as reviewed in our previous paper focusing on tomatine potential health properties [1]. These methods consume large volumes of organic solvents, have long extraction times and yield low recovery rates [12]. There is a growing need for greener extraction techniques to promote more sustainable life cycles. These green methods reduce extraction time, use less solvent and achieve higher extraction yields and better extract quality [12]. Clean and sustainable extraction techniques also ensure that the extracts are free from toxicity caused by solvent contamination [13]. Subcritical water extraction (SWE) is a promising green technique that uses water at temperatures above 100°C and pressures above its vapour pressure to maintain water in the liquid state, resembling organic solvent behaviour [14]. Under these conditions, water exhibits lower viscosity and surface tension than at room temperature, which enhances mass transfer rates. Additionally, as the temperature rises, the dielectric constant decreases due to hydrogen bond dissociation, allowing water to act as an effective solvent for moderately polar to non-polar substances. Furthermore, the ionic product of water (KW) increases with temperature, being three orders of magnitude higher than that at ambient conditions [15]. This increases the concentration of hydronium and hydroxide ions and allows water to act as an acid or base catalyst, facilitating the hydrolysis of lignocellulosic polymers and proteins to smaller oligomers and peptides, without the use of additional catalysts [16]. Examples of SWE of natural products are the extraction of polyphenols from rosemary plants [17], glycosides from *Stevia rebaudiana* leaves [18], flavonoids from ginseng leaf/stem [19] and bioactive compounds from bene hull [20] and grape pomace [16].

The purpose of this work is to assess the feasibility of using SWE to recover tomatine from green tomatoes by-product more efficiently than conventional techniques and to analyse the extracts obtained for their alpha-tomatine, carbohydrates and saponins content, total phenolic compounds and antioxidant activity, to access its viability to be used as an ingredient in pharma care.

## 2. Material and methods

### 2.1. Materials

Unripe green tomatoes were kindly provided by Italagro, S.A., Portugal. Sulfuric acid (96%), phenol (99 %), D(+)-glucose, gallic acid, vanillin, Trolox (6-hydroxy-2,5,7,8-tetramethylchroman-2-carboxylic acid), neocuproine, 2,2-diphenyl-1-picrylhydrazyl (DPPH), acetic acid, sulfuric acid, phenol, trichloroacetic acid, sodium carbonate, copper (II) chloride, ammonium acetate, ferulic acid, caffeic acid, chlorogenic acid, vanillic acid, cinnamic acid, rutin, ascorbic acid and Folin–Ciocalteu's reagents were obtained from Sigma-Aldrich (Madrid, Spain). Tomatine standard was obtained from PhytoLab phyproof® (Vestenbergsgreuth, Germany). Acetonitrile, methanol and ethanol were of high-performance liquid chromatography (HPLC) grade and were purchased from Sigma-Aldrich (Madrid, Spain). Ultrapure water was provided by reverse osmosis in a MILLI-Q System Elix® 3 from Millipore® (Massachusetts, USA). All other reagents were of analytical grade and were used without further purification.

### 2.2. Methods

#### 2.2.1. Tomatine extraction by subcritical water

**Figure 1.**
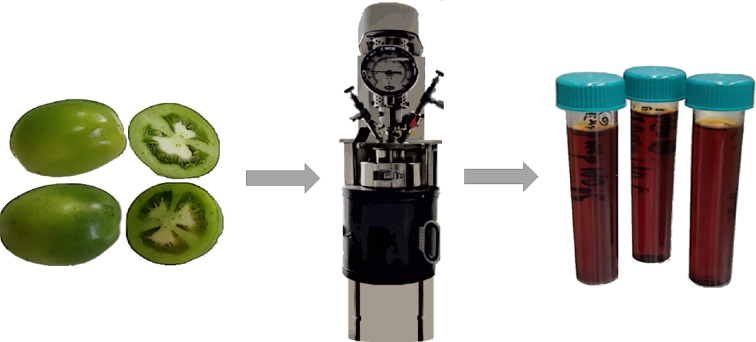
Schematic representation of SWE of tomatine from green tomatoes. From left to right: photographs of green tomatoes, the high-pressure reactor used and the macroscopic appearance of the obtained extract.

Tomatine was extracted from unripe tomatoes by SWE using a batch high-pressure reactor of 1.2 l capacity from Parr (model 4547, Illinois, USA). Different conditions of temperature (120°C and 190°C), residence time (15, 30 and 45 min) and fresh versus frozen tomatoes samples were tested. For that, the following methodology was applied. In the temperature studies, green tomatoes collected randomly by Italagro, S.A., were divided equally into two batches (A1 and A2). The tomatoes were then cut into small pieces and blended in a mixer (Kenwood, Japan). Batch A1 was used in the assay carried out at a temperature of 190°C and batch A2 was used in the assay performed at the temperature of 120°C. In both assays, a pressure of 50 bar using a nitrogen stream, a 10 : 1 solvent-to-solid ratio (chosen based on average water content of the tomato samples, *ca* 90 wt%) and a residence time of 15 min were used (figure 1). The aqueous extracts obtained were filtered through a Whatman No. 1 filter paper and centrifuged at 500*g* for 10  min at room temperature (Beckman GPR, USA; rotor GH-3.8; Beckman, Brea, CA, USA), and later stored in a refrigerator at 4°C until further use. Tomatine content of both extracts was determined by HPLC.

Next, the effect of the residence time was analysed at the temperature condition that obtained the highest extraction of tomatine. Again, green tomatoes collected randomly by Italagro were divided equally into three batches: batch B1 (15 min), B2 (30 min) and B3 (45 min), and the former procedure was followed. A pressure of 50 bar and a 10 : 1 solvent-to-solid ratio was used as before. The tomatine content in the extracts was quantified by HPLC as mentioned above.

For the fresh versus frozen assay, two batches of green tomatoes were prepared as described in the previous studies. The fresh tomatoes batch was used upon arrival (C1—fresh tomatoes). The other batch was vacuum-sealed and frozen already cut for six months before extraction (C2—frozen tomatoes).

#### 2.2.2. Physicochemical characterization of the subcritical water extracts

##### 2.2.2.1. Tomatine quantification by high-performance liquid chromatography

A previously described HPLC method [21] was used to quantify the tomatine content in the SWE extracts obtained. For comparison, an extract from green tomatoes obtained through conventional extraction with acetic acid 5% [22] was also analysed in terms of tomatine content.

##### 2.2.2.2. Fourier-transform infrared spectroscopy analysis

For Fourier-transform infrared spectroscopy (FTIR) analysis, samples were lyophilized and analysed in a Spectrum Two, Perkin-Elmer (Waltham, Massachusetts, USA), using the PerkinElmer Spectrum IR v. 10.6.2 software.

##### 2.2.2.3. Water content and Brix

Water content was calculated using an indirect method where the samples were weighed before and after lyophilization. Brix value was kindly measured by Italagro, S.A.

##### 2.2.2.4. Nitrogen content

The nitrogen content of lyophilized tomatine extracts was determined by elementary analysis. To determine the protein content, a nitrogen-to-protein conversion factor of 6.25 was used [23].

##### 2.2.2.5. Ash content

Ash content was determined using a muffle furnace [16].

##### 2.2.2.6. Carbohydrates content

Total carbohydrate content of the obtained extracts was determined by the phenol–sulfuric acid colorimetric method as described elsewhere [16], and the results are expressed as milligram of D(+)-glucose equivalent/100 g of green tomatoes (mg GE/100 g GT).

##### 2.2.2.7. Phenolic compounds analysis

Total phenolic content of the obtained green tomatoes extracts was determined by the Folin–Ciocalteu's method [24] using gallic acid (GA) as the standard. The results were expressed as milligrams of GA equivalent/100 g of green tomatoes (mg GAE/100 g GT) [16]. Liquid chromatography-Electrospray Ionization-Mass Spectrometry (LC–ESI–MS) analysis was performed for the identification of individual phenolic compounds in the C2—frozen tomatoes sample. The method used [25] had an injection volume of 1 μl and a flow rate of 0.4 ml min^−1^. The absorbance was measured at two different wavelengths, 280 and 330 nm, with a photodiode array detector. The column (Advanced Chromatography Technologies Ltd ACE 3 C18 250 × 4.6 mm (UK)) was kept at a constant temperature of 25°C. The mobile phase was a mixture of two eluents, A (0.1% (v/v) formic acid) and B (100% methanol). Gradient elution was performed as follows: the initial conditions were 10% of eluent B and then linearly increasing to 90% from 1 to 40 min, and maintaining these conditions for 5 min. The column was then returned to its initial condition at 46 min and maintained at these conditions for 10 min. The eluent was analysed using a simple quadrupole MS model 6130B (Santa Clara, CA, USA) equipped with an electrospray ionization source operating full scan over *m*/*z* 50–1000 range. The capillarity voltage was set at 3 kV with a fragmentor at 130 V. Nitrogen gas was used for MS experiments at 350°C and 12 l min^−1^. Standards of the phenolic compounds ferulic acid, caffeic acid, chlorogenic acid, vanillic acid, cinnamic acid, vanillin and rutin were prepared and analysed.

##### 2.2.2.8. Antioxidant activity

The free radical scavenging capacity of the obtained extracts was assessed by the DPPH assay [13,26]. The assay was modified according to Lisanti *et al.* [27], using Trolox as standard. Ascorbic acid was used as positive control and ethanol as negative control of the assay. Results are expressed as milligrams of Trolox equivalents/100 g green tomatoes (mg TRE/100 g GT). Total antioxidant capacity was evaluated by the cupric ion reducing antioxidant capacity (CUPRAC) assay [28], using Trolox standard. Results are expressed as mg TRE/100 g GT.

##### 2.2.2.9. Saponins content

Total saponin content was determined by the vanillin-sulfuric acid method [29,30] using tomatine standard dissolved in ethanol for the calibration curve. Total saponins are expressed as milligrams of tomatine equivalents/100 g green tomatoes (mg TE/100 g GT).

### 2.2.3. Statistical analysis

Results are presented as mean ± s.d. of two batches. For each batch, measurements were always done in triplicate. Statistical analysis was performed using GraphPad Prism 9.4.1. (GraphPad Software, San Diego, CA, USA). Unpaired *t*‐test (for two groups) or one-way analysis of variance (ANOVA) with Tukey’s test (for more than two groups) was performed, considering *p* values less than 0.05 as statistically significant.

## 3. Results and discussion

### 3.1. Subcritical water extraction of tomatine from green tomatoes

Randomized samples of 51 different tomato varieties, all collected from fields in Ribatejo, Portugal, were used to assess the influence of extraction temperature and residence time on the extraction efficiency of tomatine by SWE. There is no information available on the SWE of bioactive compounds from green tomatoes, as far as we know. Nevertheless, several authors have used subcritical water within the temperature range of 120−200°C to extract polyphenols from other natural biomasses [13,15,17,31]. Also, it is indicated that a further increase in the extraction temperature may cause the degradation of these compounds [32]. Thus, two temperatures were tested in this work: 190°C (extract A1) and 120°C (extract A2), both with a residence time of 15 min.

According to the results obtained by HPLC, no tomatine was detectable in extract A2 (table 1). The dielectric constant of water decreases sharply with increasing temperature, varying from 78.5 at 25°C to *ca* 35 at 200°C, a value that is comparable to the dielectric constant of methanol at room temperature and pressure [33]. Tomatine is insoluble in water at ambient conditions but soluble in methanol, thus corroborating the results obtained in this work. Therefore, the temperature of 190°C was chosen to study the effect of the residence time on the SWE extraction of tomatine. HPLC quantification showed no significant difference in the tomatine content among the extracts obtained with different residence times (table 1). Consequently, the shorter residence time of 15 min was selected. This result shows that tomatine is readily extracted by subcritical water. One advantage of the SWE method pointed out in the literature is its relatively fast processing time when compared to conventional extraction methods [33]. Furthermore, SWE efficiency in the extraction of tomatine from green tomatoes was significantly higher compared to conventional extraction using 5% acetic acid. One should point out that extracts A1 and B1 were obtained at similar operating conditions, i.e. 190°C, 10 : 1 solvent-to-solid ratio, and 15 min residence time, but showed different tomatine concentrations. This should be attributed to the different times of tomatoes collection of corresponding batches. The tomatoes are not always collected at the same time of maturation, so it is expected to obtain different tomatine contents.

**Table 1.** Tomatine concentration in extracts obtained by SWE and conventional method. A’s extraction was of 15 min time of residence but at different temperatures (190°C for A1 and 120°C for A2). B’s extraction was at 190°C but at different times of residence (15 min for B1, 30 min for B2 and 45 min for B3). Results are present as sextuplicate, except for the conventional extraction (triplicate). One-way ANOVA with Tukey’s test was performed, and among the batches B, there is not a statistically significant difference for a *p* < 0.05.

extract	tomatine concentration (mg alpha-tomatine/100 g green tomato)
A1	215 ± 2
A2	no tomatine detected
B1	144 ± 9
B2	145 ± 16
B3	138 ± 16
conventional extraction	70 ± 2

SWE, Subcritical water extraction.

To analyse the effect of pre-freezing the tomatoes on the extraction process, two additional assays were carried out using fresh and frozen green tomatoes at 190°C and 15 min residence time. The tomatine concentrations obtained are shown in table 2. Interestingly, the extraction yields of tomatine were comparable between the two conditions.

**Table 2.** Tomatine concentration in extracts obtained by subcritical water. Results are present as mean ± S.D. Unpaired *t*‐test was performed, and the results are statistically equal for a *p* < 0.05.

extract	tomatine concentration (mg alpha-tomatine/100 g green tomato)
C1—fresh tomatoes	175 ± 8
C2—frozen tomatoes	207 ± 1

Direct comparison between the results obtained in this study and previous studies cannot be made as this was the first time, as far as we know, SWE was applied to green tomatoes. Nevertheless, our results are in reasonable agreement with data reported in the literature for the extraction of tomatine from green tomatoes using conventional solvents [1,34]. For instance, Friedman & Levin [35] reported tomatine values for green immature tomatoes ranging from 1.6 to 17 mg/100 g of green tomatoes using aqueous solutions of acetic acid as the solvent media. On the other hand, Kozukue & Friedman [36] using a mixture of chloroform/methanol (2 : 1 v/v) obtained tomatine contents between 9.8 and 89.9 mg per 100 g fresh fruit. Choi *et al*. [37] reported values ranging from 13 to 360 mg/100 g green tomato using aqueous solutions of ethanol 80% (v/v). Although the values reported depend on the solvent media used, the species collected and respective year and degree of maturation, the range of values obtained in this work aligns well with those reported in the literature.

### 3.2. Physicochemical characterization of the extracts

**Figure 2.**
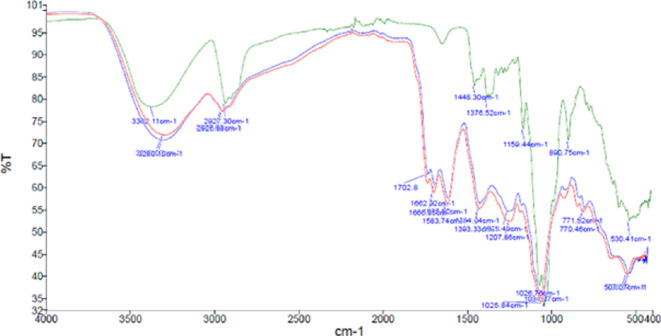
FTIR spectrums of tomatine standard (green), SWE extract C1—fresh tomatoes (red), SWE extract C2—frozen tomatoes (blue).

Samples of SWE extracts from green tomatoes were analysed by FTIR spectroscopy and compared with a tomatine standard solution. The FTIR spectrums of tomatine standard and of SWE extracts C1—fresh tomatoes and C2—frozen tomatoes are shown in figure 2.

The first valley in all spectra (around 3300 cm^−1^) is representative of –OH groups, and the second valley (around 2900 cm^−1^) is representative of the C–H group, typical of glycosylated steroidal alkaloid. The first valley in the extracts is lower than in the standard. This difference can be justified by the simple sugars present in the extract. In the extract’s spectra, the peak around 1700 cm^−1^ corresponds to carbonyl groups, typically ketone. Dehydrotomatine is probably responsible for the peak around 1500 cm^−1^ in the extract’s spectrum. Other values between 1500 and 1000 cm^−1^ are indistinct, probably corresponding to other components present in the extract. The valley around 1030 cm^−1^ represents the carbon atoms connected to oxygen atoms. This initial analysis allows us to conclude that the spectrum of the standard is similar to those of the extracts, confirming the presence of tomatine in the extract.

**Figure 3.**
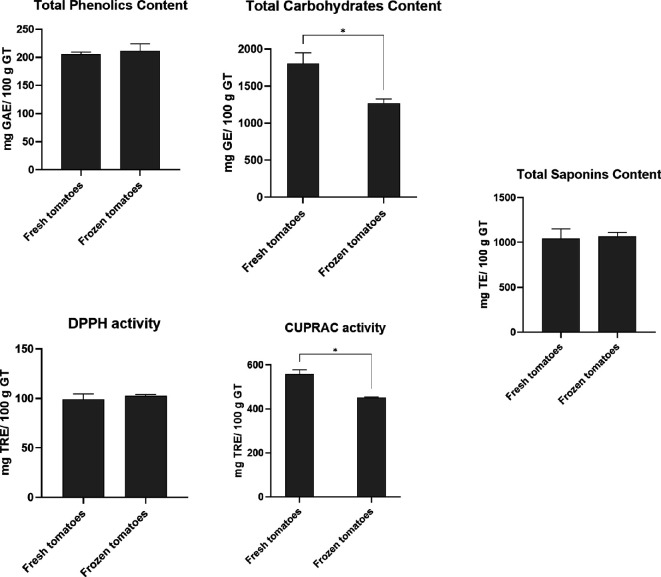
Physicochemical characterization of the extracts. Results are present as mean ± s.d. Unpaired *t*‐test was performed. A statistically significant difference is represented by * (*p* < 0.05), ** (*p* < 0.01), *** (*p* < 0.005) and **** (*p* < 0.001) when comparing all the extracts with each other. No symbol means not statistically significant. GT, green tomatoes; GAE, gallic acid equivalents; TE, tomatine equivalents; TRE, Trolox equivalents; GE, D(+)-glucose equivalents.

The SWE extracts were characterized in terms of their water and ash content, Brix value, total phenolics, protein, carbohydrates and saponins content and antioxidant activity (DPPH and CUPRAC assay). The water content was around 96% for all samples. The results obtained are shown in table 3 and figure 3.

**Table 3.** Physicochemical characterization of the SWE extracts of green tomatoes. The values without S.D. were determined by outsourcing, with no raw data available. Results are present as mean ± s.d.

extract	Brix (º)	protein (mg/100 g GT)	ash content (mg/100 g GT)
fresh tomatoes	4 ± 0	623 ± 25	736 ± 27
frozen tomatoes	4 ± 0	665 ± 21	568 ± 24

GT, Green Tomatoes; SWE, Subcritical water extraction.

The protein concentration is similar across both extracts (table 3). The total phenolic and saponins content are also equal in both extracts, with no significant difference. Regarding total carbohydrate content, there is a statistically significant difference, indicating that freezing tomatoes before extraction can affect the carbohydrate content. However, the Brix data measured does not show this difference (table 3). Brix is a measure of simple sugars, and its quantification is based on a fast assay usually used to evaluate the quality of tomatoes during tomato season in tomato processing industries [38]. The difference in the results can be related to the analytical part of the method that measures different sugars or degradation of the sugar during the freezing process.

**Figure 4.**
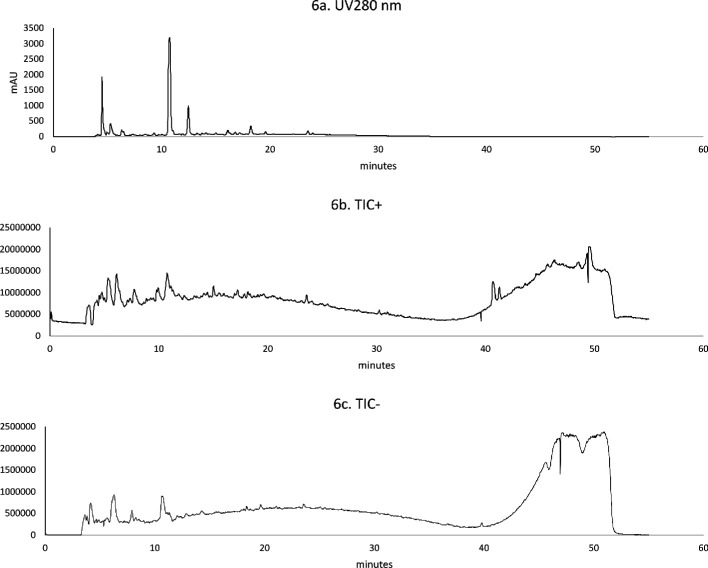
LC–ESI–MS chromatograms of SWE extract C2—frozen tomatoes: 6a. Representative UV chromatogram at 280 nm; 6b. TIC in positive mode; 6c. TIC in negative mode.

The phenolic profile of the C2 extract sample was analysed by LC–ESI–MS. Total ion chromatograms (TIC), positive and negative modes, are shown in figure 4. The chromatograms of the extract were compared with standards injected in the same conditions. A tentative attribution of peaks of compatible monoisotopic mass with compounds already described in the literature for similar extracts was also performed [22]. The analysed extract revealed the presence of ions with molecular weight compatible with molecular weights of 5-o-feruloylquinic acid, caffeoylquinic acid, p-coumaroylquinic acid and respective analogues, caffeic acid, ferulic acid, cinnamic acid, vanillic acid, vanillin and rutin, as described in table 4.

**Table 4.** LC–ESI–MS information of phenolic compounds detected in SWE extract C2.

compound	RT	*m/z*	[M − H]^−^
	(min)	[M + H]^+^	
chlorogenic acid and possible analogues (C_16_H_18_O_9_)	17.9	355	353
vanillic acid (C_8_H_8_O_4_)	19.8	—	167
caffeic acid (C_9_H_8_O_4_)	20.2	181	179
vanillin (C_8_H_8_O_3_)	21.4	—	151
ferulic acid (C_10_H_10_O_4_)	24.5	195	193
rutin (quercetin-3-o-rutinoside) (C_27_H_30_O_16_)	27.1	—	609
kaempferol 2-o-glucoside (C_21_H_20_O_11_)	a	449	—
cinnamic acid (C_9_H_8_O_2_)	32.3	149	—
*p*-coumaroylquinic acid and isomers (C_16_H_18_O_8_)	a	339	337

^a^ Not injected as a standard.

RT, Retention Time; SWE, Subcritical water extraction.

In terms of antioxidant activity, although evaluated on the same scale, the results obtained for CUPRAC and DPPH assays differ. The DPPH assay is a decolourization test that evaluates the radical scavenging activity of a compound, whereas the CUPRAC measures the ability of antioxidants to reduce Cu^2+^-neocuproine into Cu^+^-neocuproine at pH 7. The CUPRAC assay can measure the hydroxyl radical (OH) scavengers’ activity, which is the most reactive species from reactive oxygen species [39,40]. In the DPPH assays, all the extracts exhibit the same antioxidant activity. However, the CUPRAC assay results show more variability than the DPPH assay, with significant differences in antioxidant activity between extracts from fresh and frozen tomatoes.

The assessment of the antioxidant activity of tomato lipophilic extracts (from ripened tomato) and its correlation with carotenoids is extensively studied [41,42]. Direct comparison of our results with literature data is challenging because, to our knowledge, SWE from green tomatoes has not been previously performed. However, a similar study where glycoalkaloids were extracted from potato peels by subcritical water [14,43] suggested that SWE yields higher amounts of phenolic compounds and carbohydrates compared to conventional methods, which translates into higher antioxidant activity. Compared to conventional extraction from fresh tomatoes, the SWE extracts obtained in this study exhibit a phenolic content *ca* 10 times higher than those obtained by [44] using ethanol as the solvent. Consequently, the antioxidant activity of SWE extracts herein described is also superior [44]. Choi *et al*. [37] reported phenolic content ranging from 0.7 to 1.3 mg GAE/100 g GT for ethanolic extracts from green tomatoes within different maturity stages.

## 4. Conclusions

To the best of our knowledge, this is the first time SWE was used to extract tomatine from green tomatoes. The use of this green and sustainable technique allowed us to recover more tomatine than what is described in the literature, valorizing food waste. The extracts obtained through this method contain high levels of compounds with antioxidant activity. The physicochemical characterization indicates that the differences between extracts from fresh and frozen raw materials are minimal. These findings imply that green tomatoes can be available all year round, rather than being limited to the annual tomato processing season. Additionally, the characterization shows the extract’s potential for treating inflammation-associated diseases, due to its high tomatine content and other bioactive compounds. Using the extract as a whole, rather than isolating only alpha-tomatine, offers the advantage of exploiting the health-promoting properties of the various bioactive compounds present and characterized in the extract. Future work will involve *in vitro* studies to assess the extracts’ anti-inflammatory activity. Furthermore, the extracts will be incorporated into a dosage form and subsequently evaluated.

## Data Availability

The data supporting the results in this article can be accessed at the Dryad Digital Repository [45]. Should any raw data files be needed in another format, they are available from the corresponding author upon request.
